# Belimumab use, clinical outcomes and glucocorticoid reduction in patients with systemic lupus erythematosus receiving belimumab in clinical practice settings: results from the OBSErve Canada Study

**DOI:** 10.1007/s00296-017-3682-9

**Published:** 2017-03-09

**Authors:** Zahi Touma, Amyn Sayani, Christian A. Pineau, Isabelle Fortin, Mark Matsos, George A. Ecker, Andrew Chow, Sandra Iczkovitz

**Affiliations:** 1 Centre for Prognosis Studies in the Rheumatic Diseases, Toronto Western Hospital, University of Toronto Lupus Clinic, EW, 1-412, 399 Bathurst Street, Toronto, ON M5T 2S8 Canada; 2 GlaxoSmithKline Inc., Toronto, ON Canada; 3 MUHC Lupus and Vasculitis Clinic, McGill University, Montreal, QC Canada; 4 Centre de Rhumatologie, Quebec, Canada; 5 McMaster University, Hamilton, ON Canada; 6 Dalhousie University, Halifax, Nova Scotia Canada; 7 Memorial University, St. John’s, Newfoundland Canada; 8 Credit Valley Rheumatology, Canada, Mississauga, ON Canada

**Keywords:** Lupus erythematosus, systemic, B-cell activating factor, Observational study, Glucocorticoids, Disease progression, Rheumatology

## Abstract

To describe the characteristics of patients receiving belimumab, overall patterns of systemic lupus erythematosus (SLE) care, clinical outcomes, and changes in glucocorticoid dose following 6 months of therapy with belimumab, and healthcare resource utilization in belimumab users in Canadian clinical practice settings. Retrospective multicenter medical chart review study of adult patients with SLE who were prescribed belimumab as part of usual care and who received ≥8 infusions or 6 months of treatment. Primary endpoints included physician-determined overall clinical improvement from baseline, glucocorticoid use, and physician-determined SLE disease severity at Month 6. In total, 52 patients were included in the study. At belimumab initiation, 5.8/76.9/17.3% of patients had mild/moderate/severe SLE, respectively. Oral glucocorticoids were discontinued in 11.4% of patients and 59.1% received a lower dose at Month 6. At Month 6, 80.8/57.7/17.3% of patients had a physician-determined clinical improvement of ≥20/≥50/≥80%, respectively. Sixteen patients had a SLE Disease Activity Index-2K score at both baseline and Month 6, with a mean improvement of 2.6 ± 5.3 from 8.1 ± 3.2 at baseline. No formal disease assessment tool was utilized for 42.3% of study patients at baseline. This study provides the first real-world insights into belimumab use in Canada. It demonstrates significant reduction or discontinuation of glucocorticoid dose in 70.5% of patients and clinically significant improvement following 6 months’ belimumab therapy. The high number of patients with no formal disease activity assessments highlights a key care gap in SLE treatment in the real-world setting.

## Introduction/objectives

Systemic lupus erythematosus (SLE) is a chronic autoimmune disease that affects approximately 31.9–51.0 per 100,000 persons in Canada, depending on the case definition used [1]. Standard therapies for SLE include glucocorticoids, antimalarial agents, non-steroidal anti-inflammatory drugs (NSAIDs), cyclophosphamide, and immunosuppressive/immunomodulatory agents [2–6]. Despite improvements in medical care leading to improved survival of patients with SLE in recent decades [7], long-term morbidity can adversely affect patients’ quality of life and their ability to work, resulting in substantial direct and indirect costs.

Belimumab (Benlysta™) is a B-lymphocyte stimulator (BLyS)-specific inhibitor that blocks the binding of soluble BLyS, a B-cell survival factor, to its receptors on B cells [8]. Belimumab was approved in Canada in 2011 and is indicated in addition to standard therapy for reducing disease activity in adult patients with active, autoantibody-positive SLE [9]. Two pivotal Phase 3 randomized, double-blind, placebo-controlled trials (BLISS-52 and BLISS-76), conducted in adults with SLE, demonstrated superiority of belimumab over placebo plus standard of care in the primary efficacy analysis of response rate, measured by the SLE responder index (SRI) at Week 52. Reductions in disease activity and severe flares were also observed [10, 11].

While the clinical efficacy of belimumab has been demonstrated in randomized controlled trials, utilization patterns and clinical benefit in real-world clinical practice have not been studied in Canada. The OBSErve studies (evaluation Of use of Belimumab in clinical practice SEttings) are observational studies designed to describe the overall patterns of SLE care and clinical outcomes following belimumab therapy in a real-world setting in different countries. The first of the OBSErve studies to report was the OBSErve US study (GSK study 117295), conducted in the USA, which investigated the clinical effectiveness, changes in laboratory parameters and healthcare resource utilization associated with belimumab plus standard of care in a cohort of patients with SLE over 24 months in clinical practice [12]. Here we present the results of the OBSErve Canada study (GSK Study 117300), which aimed to provide real-world insights on the characteristics of patients receiving belimumab in clinical practices in Canada, as well as to describe overall patterns of SLE care, clinical outcomes, including changes in glucocorticoid dose, following 6 months of therapy with belimumab in Canadian clinical practice settings.

## Methods

### Study design

This was a multicenter, retrospective, medical chart review study conducted in Canada. The study period included the 6-month treatment history period prior to the index date (belimumab initiation date; baseline) and a 6-month follow-up period after the index date.

Rheumatologists, who managed/treated ≥10 patients with SLE, had ≥5 years of experience in treating SLE and had ≥2 patients currently receiving belimumab (of whom ≥1 patient had received ≥8 infusions) were recruited to participate in the study. Participating physicians were responsible for patient selection and enrollment, chart data abstraction and data validation. Data collection occurred by physician abstraction of patient medical chart data onto the paper-based physician practice profile form (PPPF) and study case report forms (CRFs). CRFs captured patient demographics, comorbidities, SLE disease characteristics including disease course and disease activity assessment, prior SLE treatments, belimumab initiation profile including reason for initiation, concomitant medications and healthcare resource use. All patient data were anonymized. While this study was not designed to collect data relating to safety of belimumab, nor was this an objective, available data pertaining to adverse events (AEs) were summarized and AEs that the physician deemed belimumab-related were reported to the study sponsor.

Central ethics approval for the study was obtained from the Veritas independent review board (IRB; project reference ‘Evaluation of Use of Belimumab in Clinical Practice Settings [OBSErve]’). Institutional research ethics board approvals were also obtained from the University Health Network Research Ethics Board (14-7641-BE), Hamilton Integrated Research Ethics Board (13-743-C), and McGill University Health Centre Genetics/Population Research Investigator Initiated Studies Research Ethics Board (13-097 GEN). As patient data were anonymized, a waiver of informed consent for study patients was granted by the Veritas IRB.

### Patient population

Physicians enrolled all patients from their practice who fulfilled the following inclusion criteria: adults aged ≥18 years with a diagnosis of SLE based on the physician’s judgement, who had received, as part of usual-care, ≥8 belimumab infusions (6 months of continuous treatment); reason for belimumab initiation could be identified; ≥6 months of medical history prior to belimumab initiation was documented with the participating study physician. Patients enrolled in any other SLE-related clinical trial were excluded.

### Primary study endpoints

The primary study endpoints included: physician-determined SLE disease severity (mild, moderate, severe); patterns of SLE care among belimumab users in clinical practice in Canada; SLE clinical outcome measures at Month 6, as measured by physician-determined overall clinical status compared with baseline [worse, no improvement, and improvement (<20, ≥20, ≥50, ≥80)]; and glucocorticoid use.

### Other study endpoints

Other study endpoints included measures of disease activity routinely used by physicians, such as the physician global assessment (PhGA), patient global assessment (PtGA), the Systemic Lupus Erythematosus Disease Activity Index 2000 (SLEDAI-2K) or its derivation, the British Isles Lupus Assessment Group (BILAG) score, and the Fatigue Severity Scale. The PtGA score is reported on a scale of 0 (no disease activity)−100 (high disease activity). Change in PtGA was calculated for patients who had a score recorded on the CRF at baseline and Month 6.

### Statistical analyses

Due to the largely descriptive nature of the study, no formal sample size calculations were undertaken to determine the number of physicians or patients to be included in the study. The study sample size was based solely on the number of physicians and patients who met the inclusion criteria at baseline. Descriptive statistics and univariate analyses were conducted where appropriate.

## Results

### Physician characteristics

Seven rheumatologists (four hospital-based, three community-based) were recruited. The majority (*n* = 6) practiced in urban settings. Four physicians were individual practitioners and three were members of a group practice. At the time of baseline data collection, physicians estimated that in total they were currently treating or managing a mean of 1814 ± 684.2 patients, of whom 176 ± 113.7 patients had SLE. Physicians reported a mean of 9 ± 9.3 patients currently receiving belimumab.

Four rheumatologists (57.1%) reported routinely using the PhGA and the PtGA, and two physicians (28.6%) each also reported routinely measuring SLEDAI-2K, BILAG score, and the Fatigue Severity Scale. Three rheumatologists (42.9%) reported that they did not routinely use any disease activity instrument.

### Patient characteristics

A total of 52 patients were included in the study. Patients were predominantly female (94.2%) and largely Caucasian (65.4%), followed by African origin (15.4%), Asian (11.5%), Hispanic (5.8%), and West Indian (1.9%). The mean (SD) age of the patients was 46.5 (10.8) years (Table 1). Patients had a mean of 2.8 ± 2.2 comorbidities, the most common of which were hypertension (44.2%), hyperlipidemia (23.1%), depression (15.4%), and fibromyalgia (13.5%). Approximately 40% of patients were employed (34.6% full time and 5.8% part time) and 28.8, 13.5, and 5.8% were reported as having disabled, unemployed, and retired employment status, respectively. The most frequently cited reasons for starting belimumab therapy were: previous treatment regimen was not effective (69.2%); aim to decrease use or dose of glucocorticoids (67.3%); worsening patient condition (50.0%); and previous treatment regimen not well tolerated (17.3%).

**Table 1:** Patient demographic and clinical characteristics

	Total patients with SLE (*n* = 52)	SLE disease severity		
		Mild (*n* = 3)	Moderate (*n* = 40)	Severe (*n* = 9)
Mean (SD) age (years)	46.5 (10.8)	43.3 (5.8)	46.8 (10.8)	46.7 (12.8)
Gender: female, *n* (%)	49 (94.2)	3 (100.0)	37 (92.5)	9 (100.0)
Race, *n* (%)				
Caucasian	34 (65.4)	2 (66.7)	24 (60.0)	8 (88.9)
African origin	8 (15.4)	1 (33.3)	7 (17.5)	–
Asian	6 (11.5)	–	6 (15.0)	–
Hispanic	3 (5.8)	–	3 (7.5)	–
West Indian	1 (1.9)	–	–	1 (11.1)
Time since SLE diagnosis, *n* (%)				
0–5 years	12 (23.1)	–	9 (22.5)	3 (33.3)
6–10 years	12 (23.1)	–	10 (25.0)	2 (22.2)
>10 years	28 (53.8)	3 (100.0)	21 (52.5)	4 (44.4)
SLE disease severity at baseline, *n* (%)				
Mild	3 (5.8)	3 (100.0)	–	–
Moderate	40 (76.9)	–	40 (100.0)	–
Severe	9 (17.3)	–	–	9 (100.0)
SLE disease characteristics at baseline, *n* (%)				
High anti-dsDNA	22 (42.3)	2 (66.7)	16 (40.0)	4 (44.4)
Low C4	20 (38.5)	2 (66.7)	15 (37.5)	3 (33.3)
Low C3	17 (32.7)	3 (100.0)	12 (30.0)	2 (22.2)
Leukopenia	9 (17.3)	–	8 (20.0)	1 (11.1)
Proteinuria	6 (11.5)	–	5 (12.5)	1 (11.1)
None	16 (30.8)	–	12 (30.0)	4 (44.4)
Top 5 reasons for initiating belimumab, *n* (%)				
Previous treatment regimen ineffective	36 (69.2)	2 (66.7)	27 (67.5)	7 (77.8)
Decrease use of glucocorticoids	35 (67.3)	3 (100.0)	26 (65.0)	6 (66.7)
Worsening patient condition	26 (50.0)	1 (33.3)	18 (45.0)	7 (77.8)
Previous treatment regimen not well tolerated	9 (17.3)	–	8 (20.0)	1 (11.1)
Patient request	2 (3.8)	–	2 (5.0)	–
Concomitant SLE medications, *n* (%)				
Oral glucocorticoids	44 (84.6)	3 (100.0)	32 (80.0)	9 (100.0)
Antimalarials	40 (76.9)	3 (100.0)	32 (80.0)	5 (55.6)
Immunosuppressant^a^	38 (73.1)	2 (66.7)	30 (75.0)	6 (66.7)
NSAIDs	8 (15.4)	–	6 (15.0)	2 (22.0)
Mean (SD) prednisone equivalent dose (mg/day)	13.6 (10.0)	13.3 (14.4)	13.3 (9.8)	14.7 (10.5)
Mean (SD) prednisone equivalent dose in patients with ≥7.5 mg/day at index date (mg/day)	17.8 (9.5)	–	–	–

*Anti-dsDNA* anti-double-stranded DNA, *C* complement, *NSAID* non-steroidal anti-inflammatory drug, *SD* standard deviation, *SLE* systemic lupus erythematosus

^a^Immunosuppressants included mycophenolate mofetil, azathioprine, methotrexate, cyclosporine, and mycophenolate sodium

### SLE disease characteristics

More than half of the patients (53.8%) had been diagnosed with SLE for more than 10 years (Table 1). The most common clinical manifestations were musculoskeletal (71.2%), mucocutaneous (55.8%), immunologic (30.8%), or constitutional (fatigue) (28.8%). The most frequently reported specific SLE manifestations were arthritis (69.2%), rashes (46.2%), low complement (26.9%), and increased anti-double-stranded DNA (anti-dsDNA) antibody levels (21.2%). Other associated features frequently reported were fatigue (28.8%) and alopecia (21.2%). Physician-assessed SLE disease severity, at initial SLE diagnosis and at belimumab initiation, was reported as mild/moderate/severe in 5.8/51.9/42.3% and 5.8/76.9/17.3% of patients, respectively. The majority (86.5%) of patients had ≥2 clinical SLE manifestations at belimumab initiation, with a mean of 3.1 ± 1.5 manifestations, ranging from 0.7 in patients with mild SLE to 3.2 in those with moderate or severe SLE.

In the 6 months prior to belimumab initiation, all patients received immunosuppressants, often in combination with oral glucocorticoids (96.2%), antimalarials (92.3%), and/or NSAIDs (55.8%). Combination SLE therapy continued to be common at baseline, with most patients receiving oral glucocorticoids (84.6%), antimalarials (76.9%), and/or immunosuppressants (73.1%) (Table 1). The most commonly prescribed immunosuppressants at baseline were mycophenolate mofetil (32.7%), azathioprine (21.2%), and methotrexate (17.3%).

The most frequently administered disease activity assessments at belimumab initiation among the study patient population were the PtGA (48.1%) and SLEDAI-2K (or SELENA-SLEDAI) (32.7%). The fatigue severity scale was available for 30.8% of patients, PhGA for 25.0%, and the health assessment questionnaire for 25.0%. Formal disease assessment instruments were not utilized for 42.3% (22/52) of study patients, both at baseline and Month 6.

### Clinical outcome measures

#### Overall clinical response

Following 6 months of belimumab treatment, 80.8, 57.7, and 17.3% of patients were observed by their physicians to have an overall clinical improvement of ≥20, ≥50, and ≥80%, respectively (Table 2). Nine patients (17.3%) had <20% clinical improvement and one patient (1.9%) showed no improvement; no patients were reported to have had worsened disease. Similar trends in improvement were observed across the disease severity subgroups (Table 2).

**Table 2:** Physician-determined clinical improvement from baseline at Month 6

	All patients (*n* = 52)	Mild SLE (*n* = 3)	Moderate SLE (*n* = 40)	Severe SLE (*n* = 9)
Worsened, *n* (%)	0 (0)	0 (0)	0 (0)	0 (0)
No improvement, *n* (%)	1 (1.9)	0 (0)	1 (2.5)	0 (0)
<20% improvement, *n* (%)	9 (17.3)	0 (0)	7 (17.5)	2 (22.2)
≥20% improvement, *n* (%)	42 (80.8)	3 (100.0)	32 (80.0)	7 (77.8)
≥50% improvement, *n* (%)	30 (57.7)	3 (100.0)	23 (57.5)	4 (44.4)
≥80% improvement, *n* (%)	9 (17.3)	2 (66.7)	5 (12.5)	2 (22.2)

*SLE* systemic lupus erythematosus

#### Glucocorticoid use

Of the 44 patients receiving oral glucocorticoids at baseline, five (11.4%) discontinued oral glucocorticoid therapy after 6 months of belimumab therapy (Table 3). Among patients with SLE who were still receiving oral glucocorticoids after 6 months of belimumab therapy, the glucocorticoid dose was decreased for 59.1%, remained the same for 22.7%, and was increased for 6.8% of patients (Table 3). At Month 6, the mean glucocorticoid dose had decreased by 5.8 mg/day from baseline (Table 4). The mean reduction among patients with a baseline glucocorticoid dose ≥7.5 mg/day was 8.2 ± 9.8 mg/day after 6 months of belimumab therapy (dose at belimumab initiation 17.8 ± 9.5 mg/day; dose following 6 months of belimumab 9.6 ± 6.0 mg/day).

**Table 3:** Change in glucocorticoid use from baseline to Month 6

	All patients (*n* = 44)	Mild SLE (*n* = 3)	Moderate SLE (*n* = 32)	Severe SLE (*n* = 9)
Discontinued, *n* (%)	5 (11.4)	1 (33.3)	4 (12.5)	0 (0)
Decreased, *n* (%)	26 (59.1)	0 (0)	20 (62.5)	6 (66.7)
No change, *n* (%)	10 (22.7)	2 (66.7)	7 (21.9)	1 (11.1)
Increased, *n* (%)	3 (6.8)	0 (0)	1 (3.1)	2 (22.2)

*SLE* systemic lupus erythematosus

**Table 4:** Change in oral glucocorticoid dose following 6 months of belimumab treatment

	Total patients with SLE (*n* = 52)	Mild SLE (*n* = 3)	Moderate SLE (*n* = 40)	Severe SLE (*n* = 9)
Prescribed glucocorticoids at baseline (*n*)	44	3	32	9
Mean (SD) glucocorticoid dose at belimumab initiation (mg/day)	13.6 (10.0)	13.3 (14.4)	13.3 (9.8)	14.7 (10.5)
Continued glucocorticoids at Month 6 (*n*)	39	2	28	9
Mean (SD) glucocorticoid dose at Month 6 (mg/day)	7.8 (5.8)	3.3 (2.9)	7.6 (5.7)	10.1 (6.1)
Mean (SD) change in glucocorticoid dose at Month 6 (mg/day)	−5.8 (8.9)	−10.0 (17.3)	−5.7 (6.8)	−4.6 (12.6)

*SD* standard deviation, *SLE* systemic lupus erythematosus

#### Other concomitant medications

The percentage of patients receiving antimalarials at Month 6 remained similar to baseline (78.8 vs. 76.9%), as did the percentage of patients receiving immunosuppressants (73.1 vs. 69.2%). The percentage receiving NSAIDs remained the same (15.4%).

#### SLE manifestations

In patients reported to have arthritis manifestations at baseline (*n* = 36), a clinical response of ≥20, ≥50, and ≥80% improvement in SLE disease severity following 6 months of belimumab therapy was reported in 88.9, 69.4, and 36.1% of patients, respectively. Improvements in other SLE clinical manifestations were also observed from baseline to Month 6 (Table 5); however, it is important to note that patient numbers were low in some groups.

**Table 5:** Physician-determined improvement in SLE clinical manifestations from baseline at Month 6

	Arthritis (*n* = 36)	Rash (*n* = 24)	Fatigue (*n* = 15)	Low complement (*n* = 14)	Alopecia (*n* = 11)	Increased anti-dsDNA antibody levels (*n* = 11)	Inability to taper glucocorticoids (*n* = 10)	Mucosal ulcers (*n* = 9)	Proteinuria (*n* = 7)	Leukopenia (*n* = 7)
Worsened, *n* (%)	0 (0)	2 (8.3)	0 (0)	0 (0)	0 (0)	1 (9.1)	0 (0)	0 (0)	1 (14.3)	0 (0)
No improvement, *n* (%)	1 (2.8)	3 (12.5)	1 (6.7)	4 (28.6)	3 (27.3)	3 (27.3)	1 (10.0)	2 (22.2)	3 (42.9)	5 (71.4)
<20% improvement, *n* (%)	3 (8.3)	4 (16.7)	2 (13.3)	3 (21.4)	1 (9.1)	2 (18.2)	0 (0)	0 (0)	1 (14.3)	1 (14.3)
≥20% improvement, *n* (%)	32 (88.9)	15 (62.5)	12 (80.0)	7 (50.0)	7 (63.6)	5 (45.5)	9 (90.0)	7 (77.8)	2 (28.6)	1 (14.3)
≥50% improvement, *n* (%)	25 (69.4)	12 (50.0)	4 (26.7)	6 (42.9)	6 (54.5)	1 (9.1)	4 (40.0)	6 (66.7)	1 (14.3)	1 (14.3)
≥80% improvement, *n* (%)	13 (36.1)	6 (25.0)	0 (0)	3 (21.4)	1 (9.1)	0 (0)	3 (30.0)	5 (55.6)	0 (0)	0 (0)

*Anti-dsDNA* anti-double-stranded DNA, *SLE* systemic lupus erythematosus

#### SLE disease activity assessment

Among patients with assessments at both baseline and Month 6, mean PhGA score improved by 18.4 (*n* = 11) and mean SLEDAI-2K score improved by 2.6 (*n* = 16) (Table 6). Among patients with assessments at both baseline and Month 6 (*n* = 22), mean PtGA score improved by 0.5 over 6 months.

**Table 6:** SLE disease activity scores at baseline and Month 6

	Total patients with SLE (*n* = 52)	SLE disease severity at baseline		
		Mild (*n* = 3)	Moderate (*n* = 40)	Severe (*n* = 9)
PtGA				
Total patients with score at baseline and Month 6 (*n*)	22	3	15	4
Mean (SD) score at baseline	60.5 (22.6)	46.7 (10.1)	61.4 (23.0)	67.3 (28.2)
Mean (SD) improvement from baseline to Month 6	0.5 (24.0)	0.7 (17.1)	2.6 (26.6)	−7.5 (20.4)
PhGA				
Total patients with score at baseline and Month 6 (*n*)	11	3	7	1
Mean (SD) score at baseline	39.1 (24.4)	10.3 (17.9)	49.0 (17.6)	56.0 (0.0)
Mean (SD) improvement from baseline to Month 6	18.4 (15.9)	8.3 (14.4)	26.3 (11.0)	−7.0 (0.0)
SLEDAI-2K^a^				
Total patients with score at baseline and Month 6 (*n*)	16	3	10	3
Mean (SD) score at baseline	8.1 (3.2)	4.0 (0.0)	8.4 (2.8)	11.3 (1.2)
Mean (SD) improvement from baseline to Month 6	2.6 (5.3)	0.0 (0.0)	3.0 (3.3)	4.0 (12.2)

*PhGA* physician global assessment, *PtGA* patient global assessment, *SD* standard deviation, *SELENA-SLEDAI* Safety of Estrogens in Lupus Erythematosus National Assessment-Systemic Lupus Erythematosus Disease Activity Index, *SLE* systemic lupus erythematosus

^a^SLEDAI-2K and its derivations including SELENA-SLEDAI

### Healthcare resource utilization

Over the 6 months prior to belimumab initiation, two patients (3.8%) were hospitalized a mean of 2.5 times, for a mean duration of 8.0 days; one patient had moderate SLE and the other had severe SLE. Only one (1.9%) patient visited the emergency room in the 6 months prior to belimumab initiation. Hospitalizations remained infrequent during the belimumab treatment period: one patient with severe SLE had five admissions to hospital during the first 6 months on belimumab, with a total duration of 49 days. Six (11.5%) patients had an unscheduled visit to their rheumatologist, likely for disease management, during the 6 months prior to belimumab initiation; incidence of unscheduled visits was similar during belimumab treatment [5 (9.6%) patients].

### Adverse events

AEs were reported in 12 patients (17 AEs in total); those that occurred in ≥1 patient were sinusitis/sinus infection (*n* = 3), diarrhea (*n* = 2), and cephalalgia (*n* = 2).

## Discussion

This was a non-interventional, observational retrospective medical chart review study executed in Canada. This study was commissioned to examine adult patients with SLE, receiving belimumab, in real-world clinical practice settings. The primary objective was to describe overall patterns of SLE care in adult patients with SLE prescribed ≥8 infusions of belimumab over a 6-month period, and to examine clinical status and outcomes, including glucocorticoid use.

The patient population in this real-world setting were similar to those seen in the belimumab clinical trial program in terms of manifestations, with musculoskeletal, mucocutaneous, and immunology being the most common clinical manifestations [10, 11]. In this real-world population, 42.3% of patients had elevated anti-dsDNA antibody levels at baseline. In the BLISS-52 and BLISS-76 trials, patients were required to be positive for antinuclear antibodies (ANA) or anti-dsDNA to participate. The proportions of patients with anti-dsDNA ≥30 IU/ml were slightly higher in these trials (63–77% across treatment groups) compared with the population in the present study [10, 11].

Prolonged use of high-dose glucocorticoids in SLE can cause long-term organ damage and morbidity and therefore, glucocorticoid reduction is an important factor in treatment of SLE [13]. In this study, we observed a decrease in glucocorticoid use following 6 months of belimumab therapy, with 59.4% of patients reducing their daily glucocorticoid dose and 11.4% of patients discontinuing glucocorticoid use. It should also be noted that the proportion of patients receiving glucocorticoids decreased from 96.2 to 84.6% between 6 months prior to baseline and baseline. Reductions in glucocorticoid dosage following 6 months of therapy with belimumab were more pronounced in the high glucocorticoid group (>7.5 mg/day; −8.2 mg/day) compared with all patients (−5.8 mg/day). Similar results were seen in the OBSErve US study, in which 9% of patients discontinued glucocorticoids and 77% received a lower dose at Month 6. In the OBSErve US study, the percentage of patients prescribed glucocorticoids, and the dose received, continued to decrease from Month 6 to Month 24 [12].

Regular assessment of disease activity in SLE is vital for effective treatment management, as well as to prevent the long-term consequences of persistent disease activity (e.g., organ damage, mortality, high-dose glucocorticoid use). Although both the American College of Rheumatology and the European League Against Rheumatism guidelines recommend regular monitoring of disease activity in patients with SLE [14, 15], there is no standard disease assessment tool for SLE recommended by rheumatology professional organizations. The present study found that the clinical assessment instruments most frequently used by physicians treating patients receiving belimumab in Canada were the PtGA and PhGA [used by 4/7 (57.1%) physicians each] and SLEDAI-2K, BILAG, and patient-reported outcomes on fatigue using the Fatigue Severity Scale [used by 2/7 (28.6%) physicians each]. Consistent with the findings of OBSErve US, which showed that 50% of physicians did not use any disease assessment tool [13], in this study we found that no formal disease assessment tool was utilized for 42.3% of study patients. This highlights a key care gap in the treatment of patients with SLE.

It is imperative that disease activity be measured, ideally with standardized and validated tools, prior to initiation of SLE treatments to adequately assess disease progression and patient response. The lack of use of current disease activity assessment instruments suggests an urgent need for Canadian guidelines on the assessment of patients with lupus. Rheumatologists managing other rheumatic diseases, such as rheumatoid arthritis and psoriatic arthritis, utilize standardized and validated disease activity tools (physician-derived and patient-reported outcomes) when submitting applications for biologic treatment approval and renewal. Consistent with these other biologics, many medical insurance companies require patients with SLE to have SLEDAI-2K ≥6 with positive serology (e.g., ANA, anti-dsDNA antibodies, anti-Smith antibodies, low complement) to qualify for treatment with belimumab. This could suggest that rheumatologists managing patients with lupus may be required in the future to use validated instruments for the management of patients in their clinic.

Given the absence of a standard disease assessment tool for SLE recommended by rheumatology professional organizations, the overall clinical response improvement was physician-assessed. We observed that treatment for 6 months with belimumab led to clinically significant improvements, as measured by physicians’ overall assessment, and the reduction or discontinuation of glucocorticoids. Over 80% of patients experienced a ≥20% improvement in physician-assessed disease severity and almost 60% experienced a ≥50% improvement in physician-assessed disease severity. Less than 2% of patients experienced no change in symptoms and no patients were reported by their physicians to have worsened. These results are comparable to those from the OBSErve US study, in which 88.4 and 48.7% of patients achieved ≥20 and ≥50% improvement in overall clinical response following 6 months of belimumab therapy [12]. Hospitalizations and unscheduled visits to a rheumatologist were similar and infrequent both prior to, and during, belimumab treatment.

Observational studies enable data collection from a population of patients that provide a more realistic reflection of real-world clinical practice vs. the strictly controlled patient populations included in clinical trials. In this retrospective chart review study, a major limitation was that the quantification of clinical response (primary outcome) was a non-standardized, subjective tool that has not been validated. The lack of a control group and no randomization to treatment risks bias in efficacy measurement. Nevertheless, the data show that >70% of patients were able to decrease or stop glucocorticoids, which is a quantitative endpoint, suggesting the effectiveness and tolerability of belimumab. However, the primary aim of this study was not to determine efficacy, but to establish real-world insights into the use of belimumab in clinical practice.

The requirement of at least 6 months of treatment (for 8 doses) strongly biases this study population in favor of those who responded to and tolerated belimumab treatment, or were treated by physicians who were compliant with regular patient follow-up. This may limit comparisons with other studies of belimumab use in real-world patients with SLE. Furthermore, the majority of the patients were Caucasian (65.4%), followed by of African origin (15.4%) and this may limit the generalizability of our results. Due to the number of physicians and patients participating in this study and the availability of data, some of the analyses were conducted with low patient numbers (e.g., the physician-determined improvement in SLE clinical manifestations from baseline to 6 months) and therefore should be interpreted with caution. Other limitations include that patients were included in the study based on the physician’s judgment of a diagnosis of SLE, and that information collected on the patient CRF and PPPF included only best estimates of the current SLE-treated patients and subgroups and did not equate with a complete, detailed examination and analysis of all actual records for patients with SLE at each physician’s site/practice. In addition, the CRFs are an interpretation of what was written on the patient charts and under-reporting may have occurred if not all events were recorded on the patient charts. Physicians had varying levels of experience with validated assessment instruments for SLE disease activity, and causal inference of any effect of belimumab cannot be made as the study population did not include a control arm. In this study, the majority of the patients were Caucasian (65.4%) followed by African origin (15.4%) and therefore this imposes limitation on the generalizability of our results.

This uncontrolled retrospective chart review supports the results of previous clinical trials in a real-world setting. Key findings included the reduction or discontinuation of glucocorticoids, which has important clinical implications given the adverse effects of long-term use of this therapy, and the suggestion of clinically significant improvement as measured by physicians’ overall assessment and SLEDAI-2K scores. The high number of patients with no formal disease activity assessments highlights a key care gap in the treatment of SLE in the real-world setting.
